# SupportPrim—a computerized clinical decision support system for stratified care for patients with musculoskeletal pain complaints in general practice: study protocol for a randomized controlled trial

**DOI:** 10.1186/s13063-023-07272-6

**Published:** 2023-04-11

**Authors:** Lars Christian Naterstad Lervik, Ottar Vasseljen, Bjarne Austad, Kerstin Bach, Anita Formo Bones, Fredrik Granviken, Jonathan C. Hill, Pål Jørgensen, Torbjørn Øien, Paola Marin Veites, Danielle A. Van der Windt, Ingebrigt Meisingset

**Affiliations:** 1 General Practice Research Unit, Norwegian University of Science and Technology (NTNU), Trondheim, Norway; 2 Department of Public Health and Nursing, Norwegian University of Science and Technology (NTNU), Trondheim, Norway; 3 Hallset Legesenter AS, Trondheim, Norway; 4 Department of Computer Science, Norwegian University of Science and Technology (NTNU), Trondheim, Norway; 5 School of Medicine, Primary Care Centre Versus Arthritis, Keele University, Newcastle-under-Lyme, UK; 6 Unit for Physiotherapy Services, Trondheim Municipality, Trondheim, Norway

**Keywords:** Musculoskeletal disorders, General practice, Musculoskeletal pain, Computerized clinical decision support systems, Clinical decision-making, Electronic health records, Information technology, Online systems, Biopsychosocial model

## Abstract

**Background:**

Musculoskeletal disorders represented 149 million years lived with disability world-wide in 2019 and are the main cause of years lived with disability worldwide. Current treatment recommendations are based on “one-size fits all” principle, which does not take into account the large degree of biopsychosocial heterogeneity in this group of patients. To compensate for this, we developed a stratified care computerized clinical decision support system for general practice based on patient biopsychosocial phenotypes; furthermore, we added personalized treatment recommendations based on specific patient factors to the system. In this study protocol, we describe the randomized controlled trial for evaluating the effectiveness of computerized clinical decision support system for stratified care for patients with common musculoskeletal pain complaints in general practice. The aim of this study is to test the effect of a computerized clinical decision support system for stratified care in general practice on subjective patient outcome variables compared to current care.

**Methods:**

We will perform a cluster-randomized controlled trial with 44 general practitioners including 748 patients seeking their general practitioner due to pain in the neck, back, shoulder, hip, knee, or multisite. The intervention group will use the computerized clinical decision support system, while the control group will provide current care for their patients. The primary outcomes assessed at 3 months are global perceived effect and clinically important improvement in function measured by the Patient-Specific Function Scale (PSFS), while secondary outcomes include change in pain intensity measured by the Numeric Rating Scale (0–10), health-related quality of life (EQ-5D), general musculoskeletal health (MSK-HQ), number of treatments, use of painkillers, sick-leave grading and duration, referral to secondary care, and use of imaging.

**Discussion:**

The use of biopsychosocial profile to stratify patients and implement it in a computerized clinical decision support system for general practitioners is a novel method of providing decision support for this patient group. The study aim to recruit patients from May 2022 to March 2023, and the first results from the study will be available late 2023.

**Trial registration:**

The trial is registered in ISRCTN 11th of May 2022: 14,067,965.

**Supplementary Information:**

The online version contains supplementary material available at 10.1186/s13063-023-07272-6.

## Administrative information

**Table:** 

Title {1}	**SupportPrim – A computerized clinical decision support system for stratified care for patients with musculoskeletal pain in general practice – Study Protocol for a randomized controlled trial**
Trial registration {2a and 2b}	Clinical registry: ISRCTN: 14,067,965 Ethical approval: The Regional Ethical Committee for medical and health research defined the study outside the Norwegian Health Research Act, and thus, the study does not need an ethical approval reference: REC North Norway 376,060
Protocol version {3}	27.02.2023—“1.0—SupportPrim – A computerized clinical decision support system for stratified care for patients with musculoskeletal pain in general practice – Study Protocol for a randomized controlled trial”
Funding {4}	The study was funded by the research council of Norway (ref 303,331)
Author details {5a}	Lars Christian Naterstad Lervik ^1–3^, Ottar Vasseljen^2^, Bjarne Austad^1,2^, Kerstin Bach^4^, Anita Formo Bones^2^, Fredrik Granviken^2^, Jonathan C. Hill^5^, Pål Jørgensen^2^, Torbjørn Øien^1−3^, Paola Marin Veites^4^, Danielle A. Van der Windt^5^, Ingebrigt Meisingset^2,6^ 1.General Practice Research Unit, Norwegian University of Science and Technology (NTNU) 2.Department of Public Health and Nursing, Norwegian University of Science and Technology (NTNU) 3.Hallset Legesenter AS 4.Department of Computer Science, Norwegian University of Science and Technology (NTNU) 5.School of Medicine, Primary Care Centre Versus Arthritis, Keele University 6.Unit for Physiotherapy Services, Trondheim Municipality, Trondheim, Norway
Name and contact information for the trial sponsor {5b}	The trial sponsor is the Research Council of Norway (ref 303,331)—Post@forskningsradet.no
Role of sponsor {5c}	The trial sponsor have no role in study design, collection, management, analysis, and interpretation of data; writing of the report; or the decision to submit the report for publication, including authority over these activities

## Introduction

### Background and rationale {6a}

Musculoskeletal (MSK) disorders are the main cause of years lived with disability worldwide and represented 149 million years lived with disability worldwide in 2019 [1]. The substantial financial and functional burden on the global population is thoroughly documented [1], and projections indicate that the largest contributor among MSK disorders, low back pain, will increase further in the years to come [2]. Similarly, MSK pain complaints represent every fourth patient in primary healthcare services in Norway [3] and is the diagnostic group with the highest costs to Norwegian employers and the national social security system [4]. MSK health is therefore important for human function in all aspects of life to maintain economic, social, and functional independence, as well as human capital across the life course. Most subjects with MSK pain complaints have non-specific symptoms of short duration, but many experience remission and relapse of symptoms, enduring long periods with poor health, work disability, and reduced quality of life [5, 6].

Common interventions for patients either lack documentation or at best have modest or short-term effects [7, 8]. Large inter-individual variations in symptoms, signs, and patient histories makes it difficult to adopt evidence-based guidelines in clinical setting since they are based “one-size-fits-all evidence” from clinical trials [9]. The absence of firm diagnostic evidence for common MSK pain complaints adds to the complexity and has motivated an increased interest in prognostic factors to inform patient management [8].

To address patient heterogeneity, attempts have been made to subgroup or stratify patients according to symptoms and clinical characteristics, with emphasis on prognostic and modifiable risk factors. Such subgroups may be more homogeneous where treatment can be tailored to specific characteristics of the subgroup, i.e., stratified care [10]. It is hypothesized that stratified care will improve clinical decision making, patient management, and treatment outcome, reduce overtreatment, and give better utilization of health care resources [11]. One instrument for stratified care is the Keele STarT Back Screening Tool which stratifies patients in three risk groups: low, medium, and high, based on nine prognostic factors. The risk groups are matched with treatments that target the risk profile and patient characteristics in the risk groups. The stratified care approach showed superior clinical and economic outcome compared with usual care [12]. The study also found changes in the GPs behavior, with fever referrals to secondary care for the low-risk group, and that the medium and high-risk groups were matched to treatments that better met their needs [12].

The STarT Back Tool is designed to act as a clinical decision support system, also known as patient decision aid. These tools are designed to provide clinicians and/or patients with information that may support choices about treatment or other management options [13]. Clinical decision support systems for elective surgeries have been found to improve decision quality [14, 15], knowledge [15, 16], patient satisfaction [16, 17], and health-related quality of life and reduce decisional regret and conflict [14, 15]. In addition, where treatment is overused or has capacity limitations, Clinical decision support systems have demonstrated savings in healthcare resources use and shortened waiting times for patients with greater need, leading to improved cost-efficient and cost-effective care. For example, a trial found that using CDDSs led to fewer total joint replacements and better health outcomes [15].

The stratified care approach was recently expanded from low back pain to include patients with back, neck, shoulder, or multisite pain [18, 19]. The rationale for expanding to other MSK pain presentations is that prognostic factors and evidence-based treatment options are similar across a wide range of pain presentations [20, 21]. The Keele STarT MSK Tool [22] was developed based on knowledge of generic prognostic factors across MSK pain presentations. Recently published results [23] show that the stratified care approach did not lead to superior clinical outcome but showed improved clinical decision making including more provision of written information and prescribing of over-the-counter analgesics. One of the limitations was general practitioners' (GPs) poor fidelity in using the risk tool, which could be related to the timing of using the risk tool in the first consultation, (i.e., during versus after the consultation), the high work load for GPs [24], and the time constraints in the GP consultations [25]. Thus, stratified care is still a promising approach, but current evidence suggest that generic risk assessment tools and matched treatment options need further refinement to improve effectiveness when used in patients with MSK pain complaints [26].

The Keele STarT MSK tool mainly focused on function and disability, pain and coping, comorbidity, and the impact of pain, with little emphasis on psychological and social factors, sleep, and work ability. In addition, there were issues with poor fidelity to the system among the GPs. We therefore developed a novel method to stratify patients with MSK pain complaints using latent class analysis, to include a broader biopsychosocial approach [27]. Based on 11 generic prognostic factors, we identified five phenotypes of patients irrespective of their pain presentation (neck, shoulder, back, knee, hip, and multisite pain) [28]. The five phenotypes clearly distinguished patient subgroups by type and level of symptoms, both in a developmental sample and a validation sample [28]. Furthermore, the phenotypes had different trajectories for recovery and symptoms over 1 year follow-up, indicating that the prognostic phenotyping provided a clinically meaningful subgrouping of patients with MSK pain complaints [10]. The phenotype model includes a more comprehensive coverage of prognostic factors across the biopsychosocial domain and discriminatory ability in separate samples and over time. As such, the phenotype model may prove superior to the STarT MSK tool in stratifying patients into more distinguished subgroups and, thus, improve treatment matching and treatment outcome. However, the degree of heterogeneity in patients with MSK pain complaints requires a high predictive performance of the stratification tool. To compensate for this heterogeneity, the addition of personalized treatment recommendations may further enhance the benefits of a stratified care.

To improve GPs fidelity and further refine the stratified care approach, we incorporated the phenotyping of the patients and the matched treatment options in a computerized clinical decision support system (CDSS) for GPs. In addition, we added personalized treatment recommendations based on patient-specific factors to the CDSS. In this study protocol, we describe the randomized controlled trial for evaluating the effectiveness of CDSS for stratified care for patients with common MSK pain complaints in general practice—the SupportPrim project.

### Objectives {7}

The main objective is to evaluate the effectiveness of the stratified care intervention compared to current care for patients with MSK pain complaints in general practice. By stratifying patients with common MSK pain complaints into prognostic phenotypes and targeting treatment to the different phenotypes, we hypothesize that the patients will receive more adequate care and improved outcome with reduced health care spending.

### Trial design {8}

The study will be a multicenter, cluster-randomized, controlled trial. GPs are the clusters, and each GP will be randomized to one of the two groups, i.e., current care with or without the CDSS for stratified care intervention. The allocation ratio is 1:1. The protocol is reported according to the Standard Protocol Items: Recommendations for interventional Trial statement [29].

## Methods: participants, interventions, and outcomes

### Study setting {9}

The study will be carried out in GP practices which are part of the primary health care services in Norway. The size of the GP practice can vary from a single GP to more than 10 GPs per practice. Each GP has in average 1000 patients for which he has the medical responsibility. Each patient has one regular GP. The majority of practices have several GPs.

### Eligibility criteria {10}

Inclusion criteria are age 18 or older who make appointment with their GP due to MSK pain complaints in the neck, shoulder, low back, hip, knee, or multisite pain. Patients with both acute and chronic pain can be study participants. Exclusion criteria are reduced cognitive function, specific diagnoses such as fractures, neurological conditions (i.e., stroke, multiple sclerosis, etc.), planned surgery related to the MSK pain complaint, or surgery or fracture related to the MSK pain complaint the last 6 months, active cancer disease, pregnancy-related disorders, and poor comprehension of Norwegian language. GPs participating in the study must be a part of the Norwegian publicly funded regular GP scheme (“Fastlegeordningen”).

### Who will take informed consent? {26a}

The GPs identify patients with MSK pain complaints making appointments to see their GP. The patients identified by the GP then receive a link to online information about the study with invitation to participate in the study. If patients are willing to participate, they perform self-eligibility testing and are only allowed to participate if they meet the inclusion criteria. Patients eligible for the study sign the informed consent online and automatically receives an email granting them access to the baseline questionnaires. This is a cluster-randomized controlled trial (RCT), and participants will therefore not be informed about random allocation of their GP to intervention arms. They will be asked for consent for data collection related to the study. The GPs are not involved in obtaining informed consent, the questionnaires, and consent form are available to the participants online. Finally, the GPs will check the patient’s eligibility at the first consultation, including verification that the patient’s main problem is MSK pain complaint and comprehends the Norwegian language.

### Additional consent provisions for collection and use of participant data and biological specimens {26b}

The participants are informed that data collected in the project will be used to further develop and improve the CDSS. Only anonymized data will be used in further studies, and the informed consent contains the information describing this purpose.

### Interventions

#### Explanation for the choice of comparators {6b}

This study aims to test the effectiveness of stratified care in patients with MSK pain complaints. We will evaluate if CDSS for stratified care can improve on what is currently offered to patients with MSK pain complaints; therefore, current care is the most suitable comparator. This will also allow health economic evaluation and offer answers that are of immediate relevance to health policy makers and health professionals. Furthermore, as a cluster-design is used, the use of a current care control arm avoids having to train half of the GPs and related staff and allows observation of outcomes of care as currently offered.

#### Intervention description {11a}

The intervention is described according to the TIDier checklist [30]. The experimental intervention in this study is the CDSS for stratified care where the phenotype model is integrated. Furthermore, the CDSS integrates personalized treatment recommendations and visualization of individual factors based on participant answers relevant for GPs (Additional file 1). We first describe the stratified care intervention, and then we describe the design and content of the CDSS and how the stratified care, personalized treatment recommendations, and individual participant factors are integrated in the CDSS. Finally, we describe the GPs workflow using the CDSS.

The GP will use the CDSS together with the participant and through shared decision making decide the most appropriate treatment for the participant. The CDSS with the stratified care approach provide a decision support for the GP and the participant, and the study therefore complies with the complementarity attribute of the HONcode principle [31], stating that the purpose of the information and recommendation provided in the CDSS is to support and not to replace the relationship between the participant and the GP. The GP and the participant will agree on a treatment plan, and the treatment plan will be stored and linked to the participant in a secure server at NTNU.

#### Stratified care intervention

**Fig. 1:**
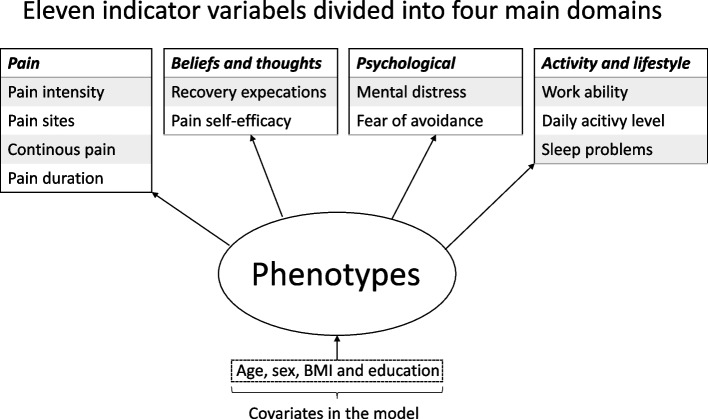
Biopsychosocial phenotypes. Eleven indicator variables divided to four main domains creates the basis for stratification of patients in to biopsychosocial phenotypes from Meisingset et al. (2020) [27]

The stratified care intervention is based on stratifying patients into five possible phenotypes according to their biopsychosocial profile. The phenotypes are derived by using latent class analysis where eleven biopsychosocial prognostic factors and four covariates are used to identify the phenotype of the patient (Fig. 1). The classification of the patient in one of the five phenotypes is integrated in the CDSS and automated using a standalone calculator with the algorithm developed by Meisingset et al. [27]. The stratified care intervention consists of providing matched treatment recommendation based on the phenotype of the patient. The details of the matched treatment recommendations are described in the “The treatment recommendation screen” section.

#### Design and content of the CDSS

**Fig. 2:**
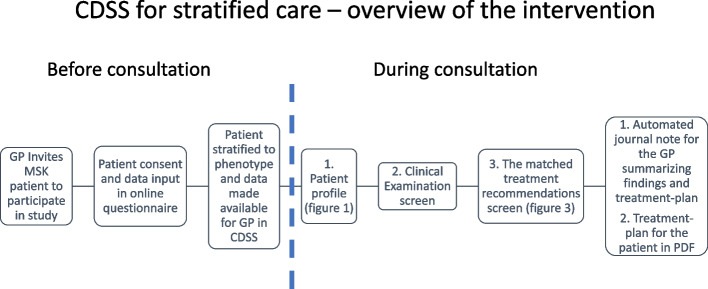
CDSS for stratified care—overview of the intervention workflow. After patient consent to participate in the study and answers questionnaires, the participants’ phenotype, a summary of the most important prognostic factors and clinical characteristics of the participant are made available in for the GP in the patient profile screen. When GP provides tentative diagnosis and data from clinical examination (optional), the matched treatment options are made available for the GP in the treatment recommendations screen. Finally, the CDSS generates a treatment plan with a summary of chosen treatment and relevant self-management literature which can be sent electronically to the patient and a journal note for the GP summarizing patient profile, clinical examination, and chosen treatment which the GP can use for the patients’ medical journal

The CDSS is designed as a standalone, computerized system with a backend hosted entirely on servers located at Norwegian University of Science and Technology. The description of the backend and the technical setup is beyond the scope of the protocol. The GPs can access the CDSS through a clinical dashboard in their web browser. The CDSS is developed based on experiences from an ongoing trial in a physiotherapy setting and adapted to GP practice. The content and the layout of the screens have been developed in close collaboration with GPs, patients, researchers, and web designers. The GPs and patients contributed to the design by reviewing “mock-ups” and suggesting placement, coloring, and important factors shown in the intervention. Furthermore, the GPs contributed with treatment suggestions and advice on how to build the automated summary of patient subjective history, clinical examination, and chosen treatment. This to adapt the CDSS to the GP workflow and to make it easy to read also for the patient as a part of shared decision making and to improve fidelity to the system among the GPs. The clinical dashboard of the CDSS consists of four main screens: (1) login and patient overview, (2) patient profile, (3) clinical examination, and (4) treatment recommendations. The CDSS for stratified care is summarized in Fig. 2.

#### Login and patient overview

The screen for login and patient overview is where the GP can log into the CDSS using their unique username and password. The screen displays the participants (with their username and birth year) that has completed the baseline questionnaire. The participant-reported data is made available for the GP immediately after the participant has completed the baseline questionnaire.

#### The patient profile screen

**Fig. 3:**
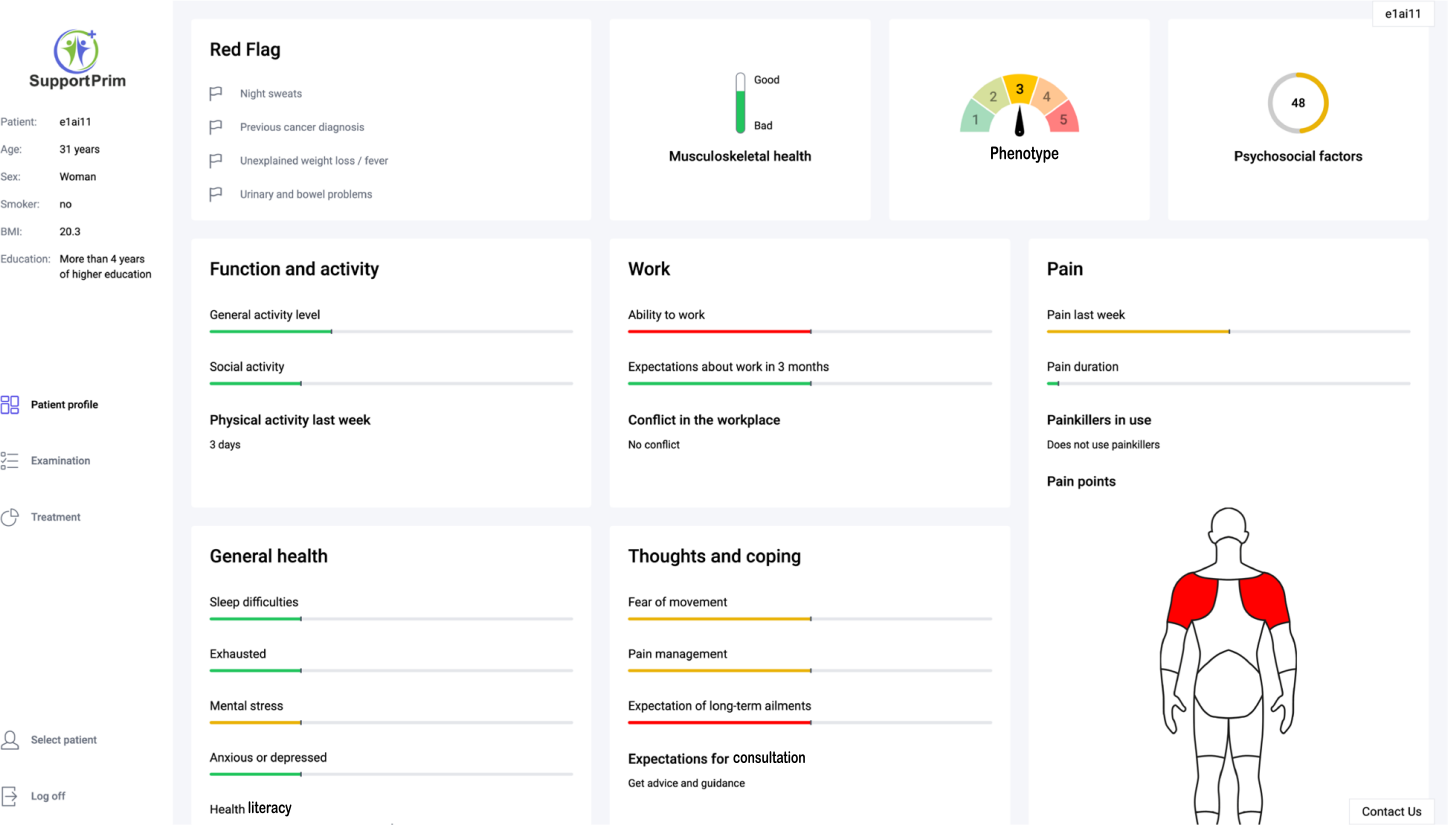
The patient profile screen. The patient profile screen displays the phenotype and a summary of the most important prognostic factors and clinical characteristics of the participant. The factors are displayed using a color system to indicate low (green), moderate (yellow), and high (red) symptom impact, which provide the GPs with a quick overview of the participant profile. The GP can use the participant profile to prepare for the consultation with the participant and as a tool to determine the focus of the consultation and to confirm the participant’s complaint

The patient profile screen (Fig. 3) displays the phenotype of the participant and a summary of the most important prognostic factors and clinical characteristics of the participant. The factors are displayed using a color system to indicate low (green), moderate (yellow), and high (red) symptom impact, which provide the GPs with a quick overview of the participant profile. The GP can use the participant profile to prepare for the consultation with the participant and to guide the conversation with the participant. Furthermore, the GP can present the patient profile to the participant and use it as a tool to determine the focus of the consultation and to confirm the participant’s complaint.

#### The clinical examination screen

The clinical examination screen is where the GP reports the main complaint (neck, shoulder, back, hip, knee, or widespread pain) and registers a few clinical tests relevant to the main complaint. Furthermore, the GP registers the ICPC-2 diagnosis, primary and secondary, if applicable. The results from the clinical examination are not integrated in the treatment recommendation provided by the CDSS.

#### The treatment recommendations screen

**Fig. 4:**
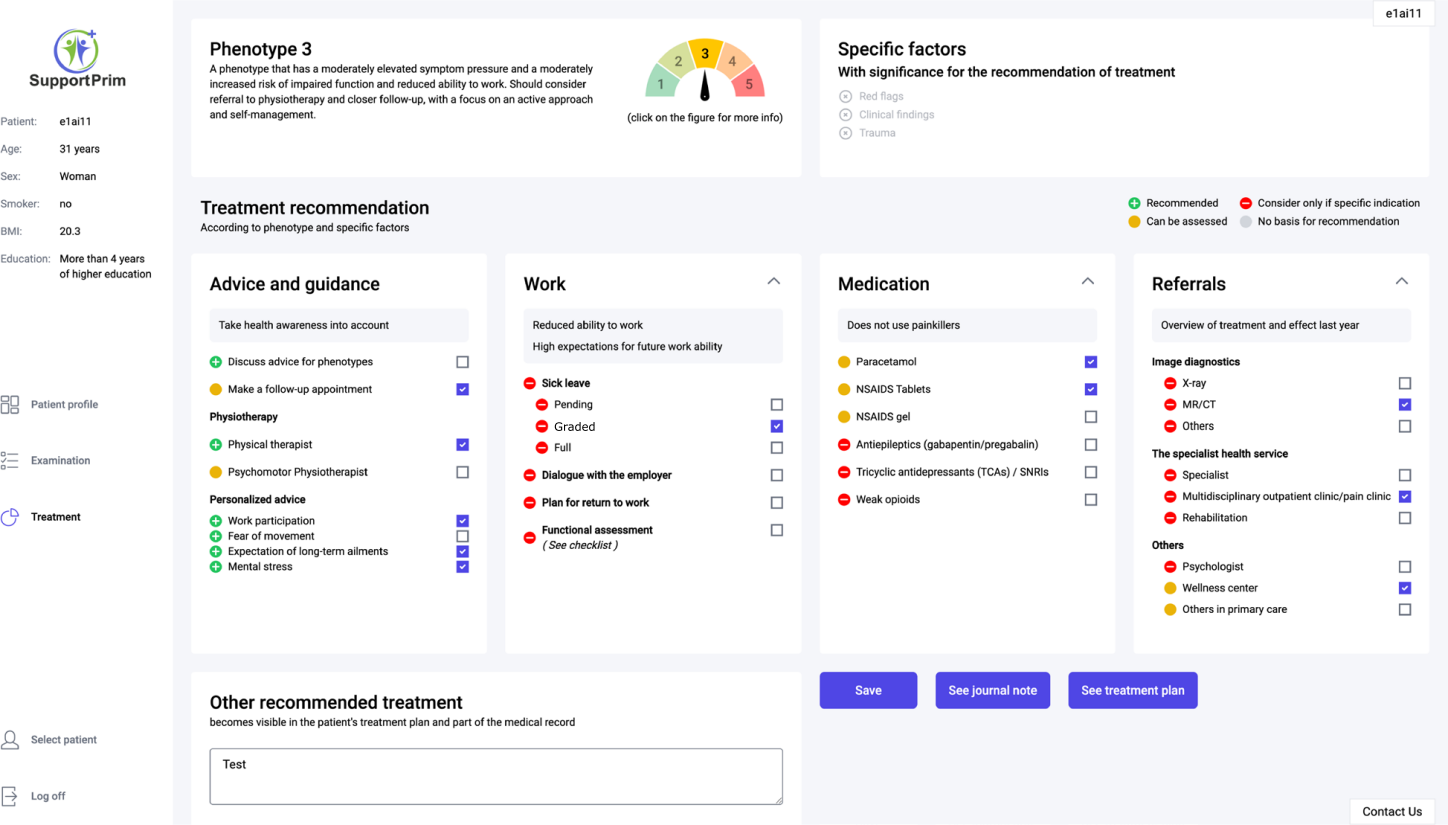
The treatment recommendation screen. The treatment recommendation screen showing general advice for the phenotype, specific factors with significance for the recommendation of treatment (red flags, clinical findings, and trauma) and treatment recommendations for advice and guidance for phenotype, personalized advice for specific patient factors, work, medication, and referrals

The treatment recommendation screen (Fig. 4) displays a description of the participant’s phenotype, participant-reported factors that directly influences clinical decision making (red flags, signs of radiculopathy in clinical examination or traumatic mechanism of MSK pain complaint), and an overview of the treatment recommendations. The recommendations are graded using a color system to indicate “recommended” (green), “can be considered” (yellow), and “consider only if specific indication” (red). The treatment recommendations are matched with the phenotype of the participant and a set of additional individual factors (Additional files 1 and 2). The recommendations are based on evidence from systematic reviews and guidelines [20] and discussions and consensus between researchers, GPs in Norway and the UK, relevant clinical specialists, and representatives from the Norwegian Labor and Welfare Administration (NAV). The treatment recommendations are categorized in four main treatment categories: (1) advice and guidance related to general and personalized advice, (2) work adaptation and sick leave options, (3) medications relevant for MSK pain complaints, and (4) referrals to primary or secondary health care and imaging. The GP will choose which treatment he will provide for the participant, and then the CDSS generates an automated text summarizing the subjective patient history and answers from the patient profile screen, clinical findings, and the treatment options chosen by the GP. The GP can then copy the text directly into the participant’s medical record. Furthermore, a treatment plan is automatically generated, which gives the participant a summary of the treatment options recommended by the GP and links to relevant self-management resources.

#### Advice and guidance

Advice and guidance matching the participant's biopsychosocial phenotype and specific participant factors constituting personalized treatment recommendations are presented as treatment recommendations for the GP. These factors include sleep disturbances, employment and work participation, osteoarthritis, smoking, weight reduction, and psychosocial factors; a complete list of advice offered is described in Additional file 1.

#### Work adaptation and sick leave options

Recommendations are provided for type of sick leave (graded, pending, or full sick leave), dialog with the employer, plan for return to work, and functional assessment. These recommendations are provided for participants reporting current employment. The recommendations differs depending on the patients expectation for future work participation assessed by the question “In your estimation, what are the chances that you will be able to work in three months’ time?”, and responded on a scale of 1–10 where 1 is not at all and 10 is extremely likely) and whether the participant reports a conflict in the workplace (Additional file 2).

#### Medication relevant for musculoskeletal pain complaints

The recommendation for use of medication was based on the NICE guidelines for treatment of MSK pain complaints, expert consensus groups, and literature review. The recommendation differentiates between phenotype 1–3 and 4–5 (Additional file 2). The rationale for this differentiation is that the duration of pain differs the most between these two groups [28] and that participants with a long-lasting pain may benefit from a different approach than participants experiencing acute pain. All recommendations are graded as “could be considered” or “only if specific indication,” i.e., yellow or red. This is because the use of painkillers in the treatment of MSK pain complaints in general should be limited and very rarely is used as sole treatment, but rather to support other treatment options, e.g., physiotherapy, exercise, and lifestyle changes [32].

#### Referrals to primary or secondary health care, and imaging

Recommendation for referral is adjusted according to participant phenotype and is described in Additional file 2. Treatment recommendations are provided for the use of imaging (X-ray, MR, or other modalities), secondary care referrals (specialist, interdisciplinary pain clinic, or rehabilitation), and other referrals (psychologist, community-based care, or others in primary care). For participants in phenotypes 1 and 2, referral to primary or secondary healthcare and imaging is recommended “only if specific indication.” For phenotype 3, referrals to community-based care or others in primary care “can be considered.” For phenotype 4, referral to secondary care and others “can be considered.” For phenotype 5, referral to secondary care and psychologist is “recommended,” and community-based health-related activities and others in primary care “can be considered.” The rationale for this differentiation is that participants with the best prognosis (i.e., lower phenotypes) should be treated in primary care and by promoting self-management, and the phenotypes with poorer prognosis can consider or are recommended referral as an option if the clinician consider it is useful for the participant.

#### GP`s workflow in the CDSS

The GPs are instructed in how to use the CDSS in their clinical practice and educated in the theoretical basis for the stratified care approach and the implications of the biopsychosocial phenotypes. The participant completes the questionnaires before the appointment with the GP. When the participant has completed the baseline questionnaires, the information is made available for the GP in the CDSS. The GPs are instructed to use the CDSS together with the participant during the consultation and involve the participant in all parts of the CDSS.

The GP starts by opening the patient profile in the CDSS. The GP can then use the patient profile to prepare for the appointment and together with the participant during the consultation. As the patient profile displays several aspects of their subjective history, including biopsychosocial phenotype, pain drawing, expectations, etc., it is recommended that the GP shows the patient profile to the patient and that they choose what to focus on in the current appointment. The GPs are also instructed to ask the participants if they feel that the patient profile properly represents their complaints.

After taking the patient subjective history and using the patient profile together with the participant, the next part of the consultation is the clinical examination. The GP opens the clinical examination screen and records the participant’s main complaint. They then perform the relevant examinations and register the findings and the primary and secondary ICPC-2 diagnosis in the clinical examination screen.

Finally, in the last part of the consultation, the GP summarizes their findings and decides on a treatment-plan for the participant based on the recommendations related to the participant’s phenotype and the personalized treatment recommendations. The GPs are instructed to use the treatment recommendations screen together with the patients to make shared decisions regarding their treatment plan. After the GP has chosen which treatment options to provide for the participant, they can generate a treatment plan containing the chosen treatment options and relevant links to self-management resources which can be sent as a PDF to the participant or printed to paper. Finally, a text summary of the patient subjective history and biopsychosocial phenotype, findings during clinical examination and chosen treatment options can be exported from the CDSS to the participant medical record at the end of the consultation. This makes it possible for the GP to work in the CDSS. As the CDSS is platform independent, i.e., available from any internet browser, it can be used with any established electronic patient journal system.

#### Control group

The control group will receive current care by the GPs randomized to this arm. Usual primary care for MSK pain complaints is known to be variable; for example, some participants may receive advice, prescriptions for medications, and nothing more, some may be asked to return to the GP for follow-up assessment or treatment, whereas others may be referred to other services, including tests and investigations, or treatment services such as physiotherapy, orthopedics, or pain clinics. Participants will answer the same questionnaires as the intervention group at all data collection time points. The GPs in the control group will have access to the login and patient overview and the clinical examination screen. The control group will not have access to the patient profile or the treatment recommendation screen.

#### Criteria for discontinuing or modifying allocated interventions {11b}

The GPs and study participants can opt out at any time point in the study. In this study, the GPs in the intervention group are exposed to a CDSS for stratified care, we do not expect adverse events from use of the CDSS as it only provides decision support for the GP, and the participant is under the GPs care. Any medical condition or change in condition will be followed up by the GP as a part of the participant’s regular care.

### Strategies to improve adherence to interventions {11c}

#### Educating the general practitioners

All GPs participating in the study will receive educating sessions individually or in small groups. Due to the widespread geographic location of the GPs, the education will be performed digitally through video conference applications. The time planned for education sessions is 60 min. The GPs will be educated in the theoretical basics for the stratification algorithm and expected recovery trajectories for the different phenotypes (only intervention), how to use the CDSS, and how to register and report data. All GPs will also receive a follow-up appointment 3–4 weeks after they start including participants. This to improve compliance with the study enrollment and recruitment of participants and to resolve any issues they might have. The education material differs between control and intervention group, and the material is also video recorded and made available for the GPs for support. We will be in regular contact with the GPs participating in the study to investigate if they experience issues with using the CDSS, if there are issues with recruitment, or if they need additional follow-up.

#### Relevant concomitant care permitted or prohibited during the trial {11d}

This is not applicable for this trial. Current care is the comparator, and the intervention is a computerized decision support system providing personalized treatment recommendations and treatment recommendations based on the participants’ biopsychosocial phenotype. As the intervention is a decision support system, its purpose being decision support, there are no restrictions in the type of treatment the GP can provide to the participants outside the CDSS recommendations.

#### Provisions for post-trial care {30}

Not applicable in this trial. Participants in this trial receive treatment from their GP and are covered by the Norwegian System of Patient Injury Compensation.

### Outcomes {12}

#### Primary outcomes

We will use two primary outcome measures:

1. The patient’s global perceived effect (GPE) at 3 months after start of treatment measured on a 7-point Likert scale. The GPE scale will be dichotomized as “improved” (scores 1–2) or “unchanged/worse” (scores 3–7). GPE is recommended as a core outcome measure in pain studies, as it may cover additional aspects to pain relief and physical function that is important to the individual [33].
2. The proportion with a clinically important improvement at 3 months in function measured by the Patient-Specific Function Scale (PSFS; 0–10). An important improvement will be defined as 30% increase on PSFS. The PSFS will also be dichotomized. Percent changes in PSFS scores will be calculated by taking the actual change in score divided by the possible change, to account for baseline values.

GPE and PSFS will also be assessed at 2, 4, and 8 weeks and included in the analysis models for the primary outcomes and presented as secondary analyses.

#### Secondary outcomes

Secondary outcomes will be change in pain intensity last week measured by the Numeric Rating Scale (0–10), health-related quality of life (EQ-5D), general MSK health (MSK-HQ), number of treatments, use of painkillers, sick-leave grading and duration, referral to secondary care, and use of imaging. We will also collect long-term outcomes at 6. Cost-effectiveness of the CDSS will be evaluated using EQ-5D at 6 months and information on treatment cost from the Norwegian Health Economics Administration. Adequacy of care (high value care) will be assessed by type and amount of treatment offered to participants in the different phenotypes. The hallmark is avoidance of overtreatment and health care spending in the phenotypes with few and uncomplicated symptoms and more comprehensive treatment for those in the phenotypes with complicated and high pain impact. Process evaluation will be carried out with semi-structured focus group interviews of GPs and participants to assess users’ acceptance and barriers and map their experience with using the stratified care intervention.

#### Participant timeline {13}

The study aims to recruit participants between 1 May 2022 and 31 March 2023. The participation timeline is further described in Table 1.

**Table 1:** Overview of participation timeline

	Study period							
	Enrolment	Baseline	Follow-up					
Timepoint week		0	2	4	8	12	26	52
Eligibility screen	X							
Informed consent	X							
Allocation	X							
Intervention								
CDSS for stratified care		X						
Assessments								
SMS			X	X	X			
Questionnaires		X				X	X	

Overview of participation timeline. As every GP is a cluster, and already allocated to intervention or control group, all participants are allocated at the time of enrolment

#### Sample size {14}

Sample size calculations based on the *clustersampsi* command in Stata for cluster-randomized, controlled trials show that 280 participants and 20 clusters (GPs) are necessary in each arm to detect a difference of 15% in proportions of participants “improved.” These calculations are based on a power of 80%, alpha level of 0.05, intra-class correlation coefficient (ICC) of 0.05, and an average cluster size of 14 participants per GP. Suggestive values for ICC were obtained from a previous project in primary care, and the same data suggest that the proportion of participants reporting “improved” after usual care physiotherapy was 50–58% in the target groups. To account for a 15–20% drop-out among the participants and 5–10% among the GPs, we will include 22 GPs in each arm and 17 participants in each cluster, giving a total of 748 participants.

Sample size calculations determined that fewer participants would be required to achieve 80% power when assessing the proportion of participants who experience a clinically important improvement in their functional status at each follow-up time point using a mixed logistic regression model, and the above sample size calculation therefore was used to determine the required number and size of clusters.

The two primary outcomes will be analyzed by multilevel modeling to account for clustering of individual data and adjust for covariates. A detailed statistical analysis plan will be published before unblinding of study data.

#### Recruitment {15}

Participating GPs will be recruited nationwide using the Norwegian Primary Care Research Network (PraksisNett, www.uib.no/praksisnett), which is a research infrastructure of close to 500 GPs that facilitates recruitment of primary care patients to clinical studies [34]. Additional GPs will be recruited through direct invitation and promotion at conferences and social media targeted at GPs.

Patients making appointments with their participating general practitioner, either online or through a phone call to the GP practice, will be informed about the study by an electronic message (i.e., SMS/Digital Dialog/Doctors office app/website/communication through Helsenorge). This is the message to the patient: “*Dr. X is collaborating with NTNU on a project to improve care for musculoskeletal pain. You can click on this link to read more about the project and decide if you want to participate*.”

Patients interested in participating will be linked to the study’s onboarding page where they can read information about the study and perform self-assessment for inclusion. If the patient is eligible, he/she is presented with an electronic consent form which contains full information about the study. If the patient consent to participation, personal data is registered (including name, phone number, and e-mail address) and a personal identification code (username) is generated. The participant is then forwarded to the electronic questionnaires and completes the questionnaires before the consultation with the GP. The username and the personal identification variables are stored in a separate secure server at NTNU. In addition, an email notification with the new participant’s name and username will be sent to the GP. The GP must have the username for the participant to identify the participant data in the CDSS.

### Assignment of interventions: allocation

#### Sequence generation {16a}

The cluster-randomization will be performed by the clinical research unit (St. Olavs Hospital and NTNU). GPs will be randomized to intervention (stratified care intervention) or control group (current care) in a 1:1 ratio. The randomization will be performed sequentially depending on the recruitment rate; initially, 22 GPs will be randomized; the remaining 22 GPs will be randomized later. GPs dropping out will be replaced by new GPs from a waiting list. The GPs recruited to the waiting list will be randomized with a ratio depending on the allocation status of the GPs dropping out from the study. The clinical research unit use a randomization procedure provided by Microsoft Excel, which is unknown for the research team in the study.

#### Concealment mechanism {16b}

The randomization is performed by a third party (The Clinical Research Unit in Central Norway) and concealed to the researchers and GPs.

#### Implementation {16c}

The randomization is performed by a third party (The Clinical Research Unit in Central Norway) and concealed to the researchers and GPs. The same party will assign the participants (GP) to their interventions.

### Assignment of interventions: blinding

#### Who will be blinded {17a}

The GPs will be unblinded to their assignment. The participants are blinded to which arm their GP is allocated to when they consent to participate in the study and completes the baseline questionnaire. The participants are not blinded after this, as the participants in the intervention participate in the shared decision-making process in using the CDSS at the first consultation. All data analyses will be performed with the cluster allocation concealed. Two researchers will do the analysis, one blinded who did not participate in the project nor in the data collection. The other one will also be blinded but has been central in administrating the data collection and will only work with data where the group allocation is concealed. The research team will secure the blinding of the analysis in collaboration with the clinical research unit involved in the trial.

#### Procedure for unblinding if needed {17b}

Not applicable as the GPs are unblinded to which arm they were allocated, and the participants are unblinded when in the consultation.

#### Plans for assessment and collection of outcomes {18a}

The data from the CDSS is stored at a secure server at NTNU. This includes all participant and GP reported data from the CDSS. The patient-reported data is collected with LimeSurvey [35]. The baseline and follow-up data in the control group is reported in the encrypted case report forms by the GP and returned to NTNU.

An overview of the questionnaires used can be found in Table 2. The online data collection forms are only available to the participants and can be made available upon request.

**Table 2:** Single questions and questionnaires to be answered by the participant at baseline and follow-up at weeks 2–52

Questionnaire	Baseline	2^a^	4^a^	8^a^	12	26	52
Demographic variables/background	X						
EQ-5D (health/life quality)	X				X	X	
Sleep and vitality item from 15D	X				X	X	
Patient specific functional scale	X				X	X	
Pain intensity (NRS)	X	X		X	X	X	
Pain mapping	X				X	X	
Workability	X	X	X	X	X	X	
Sick leave	X				X	X	
Anxiety for pain in physical activity (1 question from Tampa Scale)	X	X	X	X			
Örebro Screening Form	X						
Medication	X				X	X	
Expectation (2 questions)	X						
Received treatment last 12 months and effect of treatment	X						
Global perceived effect (1 question)		X	X	X	X	X	
Patient-therapist relationship					X	X	
Benefits and expectations to GP fulfilled?					X	X	
Adherence to treatment plan					X	X	
Most imp. reason for success/non-success					X		
Still receiving treatment from the GP?						X	
Other current diseases or ailments	X						
Red flags	X						
Description of childhood	X						
Health literacy 2 questions	X						
Physical activity HUNT	X				X	X	
HSCL-10 (emotional distress)	X				X	X	
Pain self-efficacy, 2 questions	X	X		X	X	X	
The Keele STarT MSK Tool	X						
Musculoskeletal Health Questionnaire (MSK-HQ)	X				X	X	

^a^Collected by SMS

#### Plans to promote participant retention and complete follow-up {18b}

A 24-h participant hotline is established, so the GPs (and participants) easily can contact the research group with any questions regarding the intervention or system at any time. Also, a support e-mail is established with the same purpose. We will provide a weekly newsletter for the GPs with advice for recruitment and their current recruitment status compared to the status of the other GPs in the study. We will also have weekly/bi-weekly contact with GPs through SMS and social media. Finally, we will arrange a weekly contest where the GP recruiting the most participants the previous week will receive a small prize.

To promote participant retention and completion of follow-up data, the participants’ contributions will be monitored, and participants lacking registration of 3-month follow-up data will be contacted by phone/SMS.

### Data management {19}

#### Data collection and management

Data will be obtained from the participants at baseline before the first consultation, at 3 months and 6 months follow-up. In addition, we will send some questions in weeks 2, 4, and 8 after baseline by SMS or mail. The data will be collected as follows:

1. All participant reported information at baseline and the follow-up time points will be collected using electronic questionnaires, including the SMS questions, through the survey tool LimeSurvey [14]. The participants receive a personal link to the questionnaire via mail, where they can complete the questionnaire online. A complete list of collected data is found in Table 2.
2. The GP will register information from the clinical examination and the treatment plan via the CDSS, where the stratified care intervention is completed.
3. The GP will register treatment provided at baseline and 3 months in an encrypted case report (Additional file 3).

#### Participant and clinician reported information

Table 2 describes all questionnaires and questions reported by the participant and covers demographics and different aspects related to biological, psychological, and social factors associated with pain conditions. User groups consisting of physiotherapists and GPs have been involved in the current project to decide on the questionnaires included below. The time for filling out the questionnaires at baseline is approximately 15 min. We will send a link via SMS with the questionnaires between baseline and 3 months. The GP reports the main problem, ICPC-2 diagnosis, findings from the clinical information, and provided treatment.

#### Registration of treatment prescribed by the GP

The treatment plan decided at the first consultation will be registered by the GP in the CDSS and linked to the participant in the secure server at NTNU. The treatment that was provided between first consultation and 3 months will be reported by the GPs in an encrypted spreadsheet, a “case report form” (Additional file 3).

#### Confidentiality {27}

We will use the same solution for data storage on a secure server at NTNU that was used in the SelfBack project [36] and a RCT study in physiotherapy (REC nr 49,308). The principal investigator can access the server through a username and a personal password, while the GPs can read and retrieve data from their own participants from the secure server via the CDSS. The GP have their personal username and password for the CDSS which is only linked to the GP’s own participants. The key document linking the personal data and username is stored and encrypted on a secure server separate from where the participant data is stored. Access to the key document is restricted by an encryption key, which is kept by the principal investigator. The principal investigator can grant access to the key document by providing the encryption key to other persons in the research project who is working with the data collection. The secure server is backed-up on a daily basis, and back-ups are kept for a 1-year period.

#### Plans for collection, laboratory evaluation, and storage of biological specimens for genetic or molecular analysis in this trial/future use {33}

Not applicable for this study as no biological specimens are collected.

### Statistical methods

#### Statistical methods for primary and secondary outcomes {20a}

We will publish a detailed statistical analysis plan in the ISRCTN registry. The statistical reporting will adhere to the CONSORT statement, including the extension to cluster randomized trials [37, 38]. We will describe GP characteristics as follows sex, age, location (rural/urban areas), years of working in general practice, specialist status, and number of patients under their care. We will also describe participant demographics, including sex, age, and education level to avoid selection bias.

The primary analyses for the primary and secondary outcomes will be intention to treat.

#### Analysis of the primary outcome

The effect of CDSS at 3 months will be estimated for both primary outcomes using three-level mixed logistic regression models. Each primary outcome will be assessed in a separate model that includes the repeated measures of the outcome at 2 weeks, 4 weeks, 8 weeks, and 3 months follow-up as the dependent variable and such that they are clustered by follow-up timepoint (level 1), participants as level 2, and GPs as level 3. Treatment allocation, time point, and an interaction between treatment allocation and time point will be included as independent variables. We will adjust for the stratification variable and possible prognostic variables (age, sex, education, and pain-duration). In addition, when analyzing PSFS, we will adjust for the baseline value.

The treatment effect will be estimated for each time point from the mixed logistic regression models and presented as an OR with 95% confidence intervals.

#### Analysis of secondary outcomes

Continuous secondary outcomes assessed at multiple follow-up time points will be assessed using linear mixed models. As for the primary binary outcomes, the repeated measures of each outcome at baseline, 2 weeks, 4 weeks, 8 weeks, and 3 months follow-up as the dependent variable, with follow-up timepoint as level 1, participants as level 2, and GPs as level 3. Timepoint and an interaction between treatment allocation and time points will be included as independent variables. Treatment allocation will not be included as a separate main independent variable as recommended by Twisk et al. (2018).

Continuous and binary outcomes measured at baseline and once during the follow-up period will be analyzed using linear or logistic regression, respectively. For each outcome, the follow-up measurement will be included as the dependent variable, with treatment allocation as the primary independent variable of interest. These analyses will also be adjusted for the baseline value of the outcome variable and possible prognostic variables (e.g., age, sex, education, and pain-duration). These analyses will include only participants who have completed both the baseline and 3-month follow-up questionnaires. We have not planned any imputation of missing data for these secondary outcomes.

Details of the analyses will be published in the statistical analysis plan. Stata/MP v17.0 will be used to all statistical analyses (College Station, Texas, USA).

#### Interim analyses {21b}

Not applicable in this study as interim analysis will not be performed.

#### Methods for additional analyses (e.g., subgroup analyses) {20b}

For exploratory analysis, we hypothesize that GPs might use the CDSS in a better way when they have used the system for a while and those having higher uptake with the CDSS might have larger effect with participants. Complex participants, with more pain sites, higher severe symptoms, and/or poorer prognosis might have larger effect of the CDSS. Therefore, we will do exploratory analysis of these pre-specified subgroups:

- The first 9 recruited participants compared to the participants numbered 10 and above for each GP
- Participants with two or less MSK pain sites versus participants with 3 or more MSK pain sites at baseline
- Participants with less than 1.85 points versus participants with 1.85 points or higher in Hopkins Symptom Checklist (HSCL-10) at baseline
- Participants with scores < 8 vs ≥ 8 on the work ability scale at baseline
- Based on user data of the CDSS (clicks in the CDSS); GPs with number of clicks in the lower vs the upper quartile in the intervention group to see if higher uptake of the CDSS is important
- Comparing effectiveness in the five phenotype groups as defined by our previous work [27]

We will carry out exploratory subgroup analyses to assess for treatment effect heterogeneity for each of the subgroup categorizations defined above. For these analyses, separate mixed logistic regression models will be extended to include each subgroup and an interaction term between treatment allocation and the subgroup. The estimated treatment effect will be estimated and reported for each subgroup. The interaction term will be used for assessing the presence of treatment effect heterogeneity where the comparison is between two subgroups. For the assessment of treatment effect heterogeneity based on the five phenotype groups, overall treatment effect heterogeneity will be assessed based on a likelihood ratio test comparing the models with and without the interaction term. Comparison of treatment effect between phenotype pairs will be considered if the likelihood ratio test indicates an overall treatment effect heterogeneity.

These analyses are considered to be exploratory as the trial was not powered for subgroup analyses. The results from all subgroup analyses will be presented in either the manuscript or supplementary files.

#### Methods in analysis to handle protocol non-adherence and any statistical methods to handle missing data {20c}

The analysis of both primary outcomes will use mixed logistic regression models, incorporating all available data from each participant with at least one follow-up measurement. This method should provide unbiased estimates of the effect of CDSS system under the assumption that the missing data is missing at random (MAR). We have not planned to use any other strategies handling missing data, such as multiple imputations, as this would also depend on the MAR assumption.

Number of participants with missing data for each outcome at each timepoint will be presented as well as a presentation of baseline characteristics of the full sample alongside those included in the primary analysis.

#### Plans to give access to the full protocol, participant-level data, and statistical code {31c}

This study protocol, including the prespecified and published statistical analysis plan, covers all aspects of the trial. We will publish the statistical code in the manuscripts.

### Oversight and monitoring

#### Composition of the coordinating center and trial steering committee {5d}

The coordinating center consists of two employees from the medical and technical research groups. The roles will be handling the day-to-day operations in the trial, following up feedback from participating GPs/participants to relevant personnel and keep in contact with the participating GPs. The coordinating center will report to the research group with feedback and issues during the study.

#### Composition of the data monitoring committee, its role and reporting structure {21a}

The data monitoring committee consists of two persons not involved in the day-to-day operations in the trial. The committee will monitor the progress of the data collection and secure the safety of the data and report to the principal investigator if changes in the trial are needed.

#### Adverse event reporting and harms {22}

We expect no adverse events or harms related to participating in the trial, since all treatment recommendations provided by the CDSS are part of current care in general practice. In addition, the CDSS provide decision support and will not replace the responsibility of the GP to provide safe and evidence-based treatment for the participant.

#### Frequency and plans for auditing trial conduct {23}

The trial will be conducted according to NTNU’s guidelines which encompass the possibility of an internal quality-assuring control where all aspects of the trial may be audited (protocol adherence, data management systems, formal approvals, etc.). This procedure is thus independent from the investigators and the trial sponsors.

#### Plans for communicating important protocol amendments to relevant parties (e.g., trial participants, ethical committees) {25}

All trial changes and amendments will be reported to the ethical committee and added to the trial registry ISRCTN. Participating GPs will be informed by email or phone if important changes are done during the trial.

#### Dissemination plans {31a}

Publications will include a study protocol paper and papers on effects on primary and secondary outcomes of the stratified care intervention, and cost-effectiveness and health care service spending will also be reported. Papers reporting the results from the RCT will adhere to the CONSORT guideline for reporting of results from RCTs. User acceptance and GPs experiences with using the stratified care intervention will be described in a qualitative paper. Papers will be published in international peer reviewed journals, preferably open access. Results from the study will be presented at relevant national and international conferences, and social media (Twitter, blogs, etc.) will be used to disseminate the findings to the wider public.

## Discussion

This trial will assess the effect of a stratified care CDSS on GPE and PSFS in participants with MSK pain complaints in general practice. Furthermore, the study will investigate as secondary outcome measures, avoidance of overtreatment. The ongoing crisis in the primary healthcare sector in Norway particularly affects the GPs. This means that for this to be an effective study it has to prove valuable for the GPs. We tried to emphasize this during development of the CDSS and create a system which could help the GPs handle their MSK patients more efficiently; however, there is a risk that the current workload on the GPs are overwhelming and that this may affect recruitment rates both for participating GPs and patients negatively.

## Trial status

Protocol version 27.02.2023—1.0—SupportPrim—a computerized clinical decision support system for stratified care for patients with musculoskeletal pain in general practice—study protocol for a randomized controlled trial.

## Supplementary Information

**Additional file 1.** Personalized treatment recommendations.

**Additional file 2:**. Treatment recommendations based on participant phenotype. **Table 1.** Advice according to patient phenotype. **Table 2.** Advice and guidance – stratified care recommendations for treatment and follow-up based on patient phenotypes. The recommendations are graded using a color system to indicate “recommended” (green), “can be considered” (yellow), and “consider only if specific indication” (red). **Table 3.** Overview over recommendations provided for sick leave in the treatment screen. **Table 4.** Treatment recommendations for medication provided in the treatment screen. **Table 5.** Treatment recommendations based on patient phenotype for referrals (imaging, secondary care and others) in the treatment screen.

**Additional file 3.**. Case report form.

## Data Availability

The research group conducting the study will have access to the final trial dataset. There are no contractual limitations for such access for investigators. The data will be made available upon reasonable request.

## Abbreviations

CDSS: Computerized clinical decision support system
EQ-5D: Health-related quality of life
GP: General practitioner
GPE: Global perceived effect
HSCL-10: Hopkins Symptom Checklist
ICC: Intra-class correlation coefficient
ICPC-2: International Classification of Primary Care, 2nd edition
MAR: Missing at random
MSK: Musculoskeletal
MSK-HQ: Musculoskeletal Health Questionnaire
NAV: Norwegian Labor and Welfare Administration
NTNU: Norwegian University of Science and Technology
PSFS: Patient-Specific Function Scale
SMS: Short Message Service
