# Supplementary material for: SupportPrim—a computerized clinical decision support system for stratified care for patients with musculoskeletal pain complaints in general practice: study protocol for a randomized controlled trial
Source: Trials. 2023 Apr 11;24:267. doi: 10.1186/s13063-023-07272-6 (PMC10088189; doi:10.1186/s13063-023-07272-6)
Supplement: Supplementary file 1 — Additional file 1. Personalized treatment recommendations. [file 13063_2023_7272_MOESM1_ESM.docx]

Appendix 1 – personalized treatment recommendations

| **Advice and guidance** | **Question** | **Answer / score triggering advice** | **Advice provided for the GP** |
| --- | --- | --- | --- |
| Advice on psychosocial factors | Örebro score | ≥ 50 | Map psychosocial factors such as stress, tension, depressed mood, sleep and expectations of long-term ailments and work participation, Can the total load be changed? |
|  |  |  | Comorbidities? |
|  |  |  | The need for contact with delegates in the workplace, interdisciplinary cooperation or further referral if there is a risk of permanent work reduction or disability |
| General and physical activity level | How reduced is your daily activity because of pain or problems? | Very reduced | All forms of activity are better than nothing, physical activity is good for your health. |
|  |  | Quite reduced | Discuss with the patient options for increasing the level of activity in everyday life. Make a plan |
|  | MSK HQ Question 15 - How many days of the past week have you been physically active for 30 minutes or more? | 0 or 1 day |  |
| Social activity | How much have your joint or muscle symptoms interfered with your social activities and hobbies in the last 2 weeks? | Severly reduced | Important to map barriers and opportunities for social activities, discuss with the patient, and make a concrete plan to increase social activity |
|  |  | Extremely | Important to bond and participate in the workplace |
| Sleep | QUALITY OF LIFE QUESTIONNAIRE (15D©) – question 5. Sleeping | I have moderate problems with sleeping, e.g. disturbed sleep, or feeling I have not slept enough | Does the patient have any idea of the cause of the sleep problems? |
|  |  | I have great problems with sleeping, e.g. having to use sleeping pills often or routinely, or usually waking at night and/or too early in the morning | What measures has the patient tried? |
|  |  | I suffer severe sleeplessness, e.g. sleep is almost impossible even with full use of sleeping pills, or staying awake most of the night. | Discuss sleep-hygiene advice with the patient (screen use, relaxation, physical work and exercise, adequate sleep(7-8hours), routines for falling asleep, sleep and wakefullness all days, sleep depriviation etc |
| Exhaustion | QUALITY OF LIFE QUESTIONNAIRE (15D©) – question 14. Vitality | I feel moderately weary, tired or feeble | Map the patients total load in everyday life and work |
|  |  | I feel very weary, tired or feeble, almost exhausted | What is important to prioritize everyday life and how to balance activity and rest |
|  |  | I feel extremely weary, tired or feeble, totally exhausted. |  |
| Anxiousness or depression | EQ-5D Depression | I am moderately anxious or depressed | Is the main problem depression or anxiety? |
|  |  | I am severely anxious or depressed | Does the patient have any idea of the cause of the anxiety/depression? |
|  |  | I am extremely anxious or depressed | Consider further referral or interaction with other healthcare personnel in cases of high levels of anxiety and/or depression |
| Psychic stress | Hopkins Symptom Checklist-10 | ≥ 1.85 | What is the patient's opinion on the causes related to the mental strain? Consider further referral or interaction with other healthcare personnel in the event of a high degree of mental strain |
| Fear of movement | Örebro Musculoskeletal Pain Screening Questionnaire (Short) - I should not do my normal work with my present pain. | 5-10 | Map fear, whether it is rational or irrational Gradual exposure to fear, important to address specific movements one is afraid of and not general movement. If not specific fear, adapted general training and activity to lessen fear may make sense |
| Pain with focus on prognosis | Örebro Musculoskeletal Pain Screening Questionnaire (Short) - How would you rate the pain that you have had during the past week? | 6-10 | Explain simple pain physiology (important sense/warning system that we are all equipped with) |
|  | Örebro Musculoskeletal Pain Screening Questionnaire (Short) - How long have you had your current pain problem? | >3 months | Acute vs chronic pain (when the pain sensation is appropriate and when it loses its value) |
| Pain management | Pain Self-efficacy Questionnaire | 0-5 | Focus on the strengths of the patient, what the patient achieves. Make exercises so simple that the patient experiences mastering the task |
| Expectiation of chronicity | Örebro Musculoskeletal Pain Screening Questionnaire (Short) - In your view, how large is the risk that your current pain may become persistent? | 5-10 | Discuss expectations for treatment, prognosis and course. What information does the patient bring with them from other healthcare professionals or others, discuss any conflicting information? |
| Work participation | Compare your work ability to when it was at the peak in your life. A score of 10 equals your best work ability. How would you rate your current work ability? | 0-5 | Discuss total load, rest, and exercise. Harmlessness of symptoms and ailments, reassurance. Assess the need for interaction with the employer, other therapists, occupational health services, NAV, others. |
|  | What is your expectation for todays appointment with your GP? | Sickleave |  |
|  | What is your current state of employment? | On sickleave |  |
|  | Örebro Musculoskeletal Pain Screening Questionnaire (Short) - In your estimation, what are the chances you will be working your normal duties in 3 months | 0-5 |  |
| Conflict at work | Are you experiencing a conflict at work? | Yes | Conflict in the workplace is not grounds for sick leave, but a matter between the employee and the employer. |
|  |  |  | Dialogue with the patient about the cause and what the patient sees as a possible solution. |
|  |  |  | Encourage patients to engage in dialogue with their employer through pending sick leave. |
| Weightloss | Body Mass Index | > 30.0 | Is the patient motivated for lifestyle change and weight reduction? |
|  |  |  | What has been tried in the past? |
|  | If the patient reports osteoarthritis | > 25 | Osteoarthritis knee or hip? |
| Arthrosis | Do you have osteoarthritis? | Yes | According to national and international guidelines, all persons with symptoms and findings of osteoarthritis of the hip and/or knee should be offered basic treatment consisting of information, exercise and advice on weight reduction (if necessary). |
| Smoking cessation | Do you smoke? | Yes | Is the patient motivated? |
