# Supplementary material for: SupportPrim—a computerized clinical decision support system for stratified care for patients with musculoskeletal pain complaints in general practice: study protocol for a randomized controlled trial
Source: Trials. 2023 Apr 11;24:267. doi: 10.1186/s13063-023-07272-6 (PMC10088189; doi:10.1186/s13063-023-07272-6)
Supplement: Supplementary file 2 — Additional file 2:. Treatment recommendations based on participant phenotype. Table 1. Advice according to patient phenotype. Table 2. Advice and guidance – stratified care recommendations for treatment and follow-up based on patient phenotypes. The recommendations are graded using a color system to indicate “recommended” (green), “can be considered” (yellow), and “consider only if specific indication” (red). Table 3. Overview over recommendations provided for sick leave in the treatment screen. Table 4. Treatment recommendations for medication provided in the treatment screen. Table 5. Treatment recommendations based on patient phenotype for referrals (imaging, secondary care and others) in the treatment screen. [file 13063_2023_7272_MOESM2_ESM.docx]

# Appendix 2 – treatment recommendations based on participant phenotype

Table 1: Advice according to patient phenotype

| **Phenotype** | **Advice** |
| --- | --- |
| Phenotype 1 | A phenotype characterized by low pain impact and a good prognosis, it is therefore important to have a strong focus on self-management and provide little treatment. |
| Phenotype 2 | A phenotype that has somewhat elevated pain impact, but has a good prognosis. Can consider short-term follow-up in the primary health service, but only to ensure self-management. |
| Phenotype 3 | A phenotype that has moderately elevated pain impact and a moderately increased risk of reduced function and reduced ability to work. Referral to physiotherapy and closer follow-up should be considered, with a focus on an active approach and self-management. |
| Phenotype 4 | A phenotype that has moderately elevated pain impact, complex ailments and a medium/high risk of impaired function and long-term ailments. Should consider referral to interdisciplinary follow-up/psychologically informed treatment in the primary health service with a focus on psychosocial factors. |
| Phenotype 5 | A phenotype that has high pain impact and significantly impaired function and ability to work. High risk of long-term ailments and functional impairment. It is important to have close and mainly interdisciplinary and psychologically informed follow-up with a focus on psychosocial factors. Can consider referral for interdisciplinary follow-up in secondary care (pain clinic / outpatient clinic physical medicine). |

Table 2: Advice and guidance – stratified care recommendations for treatment and follow-up based on patient phenotypes. The recommendations are graded using a color system to indicate “recommended” (green), “can be considered” (yellow), and “consider only if specific indication” (red).

|  | Phenotype | | | | |
| --- | --- | --- | --- | --- | --- |
| **Advice and guidance** | 1 | 2 | 3 | 4 | 5 |
| Discuss advice for phenotypes |  |  |  |  |  |
| Consider follow-ups |  |  |  |  |  |
| **Physiotherapy** |  |  |  |  |  |
| Physiotherapist |  |  |  |  |  |
| Psychomotoric physiotherapist |  |  |  |  |  |

Table 3: Overview over recommendations provided for sick leave in the treatment screen.

|  | **Örebro question 8** | | | **Örebro question 8**  **AND reported conflict at work** | | | |
| --- | --- | --- | --- | --- | --- | --- | --- |
|  | 0-5 | 6-7 | 8-10 | 0-5 | 6-7 | 8-10 |  |
| **Sick leave** |  |  |  |  |  |  |  |
| - Preliminary/to be decided |  |  |  |  |  |  |  |
| - Graded |  |  |  |  |  |  |  |
| - Full |  |  |  |  |  |  |  |
| **Dialogue with employer** |  |  |  |  |  |  |  |
| **Plan for return to work** |  |  |  |  |  |  |  |
| **Functional assessment** |  |  |  |  |  |  |  |

The recommendations are provided for type of sick leave (graded-, pending- or full sick leave), dialogue with the employer, plan for return to work and functional assessment. The recommendations differs depending on the patients expectation for future work participation assessed by the question “In your estimation, what are the chances that you will be able to work in three months’ time?” (Örebro question 8) and responded on a scale of 1-10 where 1 is not at all and 10 is extremely likely) and whether the participant reports a conflict in the workplace. The recommendations are graded using a color system to indicate “recommended” (green), “can be considered” (yellow), and “consider only if specific indication” (red).

Table 4: Treatment recommendations for medication provided in the treatment screen.

| **Phenotype** | **1** | **2** | **3** | **4** | **5** |
| --- | --- | --- | --- | --- | --- |
| Paracetamol (21) |  |  |  |  |  |
| NSAIDS (41) |  |  |  |  |  |
| Topical NSAIDS (42, 43) |  |  |  |  |  |
| Antiepileptic medication (gabapentin/pregabalin) |  |  |  |  |  |
| Tricyclic antidepressants or serotonin-noradernalin reuptake inhibitors |  |  |  |  |  |
| Weak opioids |  |  |  |  |  |

The recommendations are graded using a color system to indicate “recommended” (green), “can be considered” (yellow), and “consider only if specific indication” (red).

Table 5: Treatment recommendations based on patient phenotype for referrals (imaging, secondary care and others) in the treatment screen.

| **Phenotype** | **1** | **2** | **3** | **4** | **5** |
| --- | --- | --- | --- | --- | --- |
| **Imaging** |  |  |  |  |  |
| - X-ray |  |  |  |  |  |
| - Magnetic resonance imaging/CT-scan |  |  |  |  |  |
| - Other modalities |  |  |  |  |  |
| **Secondary care** |  |  |  |  |  |
| - Specialist |  |  |  |  |  |
| - Interdisciplinary pain clinic |  |  |  |  |  |
| - Rehabilitation |  |  |  |  |  |
| **Others** |  |  |  |  |  |
| - Psychologist |  |  |  |  |  |
| - Community based health related activities |  |  |  |  |  |
| - Others in primary care |  |  |  |  |  |

The recommendations are graded using a color system to indicate “recommended” (green), “can be considered” (yellow), and “consider only if specific indication” (red).
